# Certain Interesting Points in the Treatment of Eruptive Diseases

**Published:** 1898-12

**Authors:** James G. Wilson

**Affiliations:** Professor of Medicine Jefferson Medical College


### Southern Medical Record.

A MONTHLY JOURNAL OF MEDICINE AND SURGERY.

VOL. XXVIII. ATLANTA, GA., DECEMBER, 1898. NO. 12.

### Original Articles.

### CERTAIN INTERESTING POINTS IN THE TREATMENT OF ERUPTIVE DISEASES.

By JAMES G. WILSON, M.D.

Professor of Medicine Jefferson Medical College.

#### THE EXACT PERIOD OF INCUBATION IN MEASLES.

The following case, which is that of a man who was admitted to the hospital on September 20, 1898, suffering from enteric fever, presents several interesting features over which we will spend a few minutes in study. Before taking up the discussion of the point for which I bring him before you today, let me say a few words on the question of the "military treatment" of enteric fever, which is a point that has attracted some attention from the public and the medical profession lately. This man is a soldier who was in the fourteenth day day of his disease when we admitted him to the hospital; he had a high fever, reaching above 104 degrees, for which he was treated by systematized cold baths, which were not especially satisfactory in his case. It has been the belief of the early advocates of this system of bathing, that to be effective the baths must begin early in the course of the disease. Some authorities even have gone so far as to state that, if begun promptly on the outset of the trouble, there should be no deaths at all; this is, of course, extreme, still it serves to illustrate our point. Brandt in the Franco-Prussian war had the opportunity to study eleven hundred cases in which the treatment was begun early. This is much more possible in military service than in civil life, for under the plan of daily military inspection, a soldier could be treated with bathing from the very first day of his illness, because his sickness would be called at once to the attention of his physician, especially if there was an epidemic of enteric fever prevailing; whereas, in private practice, or even in hospital work, generally four or five days, at least, pass before the doctor is consulted, and then these patients come with the complaint that they have bilious attacks and some more days are consumed in making a diagnosis. Hence, much of the value of cold bathing in enteric fever comes from its prompt and early application. This man has had nineteen baths in all; some of these reduced his temperature seven and eight degrees at a time.

But the most interesting thing about the case and the reason I bring him before you, is that he states that he was placed in an ambulance with a man suffering with measles, which he himself had never had. As a result he got another infection which ran its course independently of the enteric fever. It is difficult generally to fix definitely the period of incubation in these infective diseases, but here is a case in which the time of exposure is definitely known. On the thirteenth day after exposure the first symptoms of measles were noticed, and on the fifth day following the rash appeared, or eighteen days after the first exposure. In the text-books the time is variously stated from seven to fifteen days; this case, taking the weakened condition of the patient into consideration, points to the longer period as being the most likely. As to the character of the rash, it is my belief that the character of the skin determines its intensity rather than the severity of the disease; in cases where the skin is of a sensitive, easily irritated nature the eruption is more marked than in less susceptible integuments.

THE TREATMENT OF SHINGLES.

Our next patient represents another type of eruptive disease, in which we have before us a typical example. This boy, fourteen years of age, has over the lower surface of his ribs on the left side a sharply marked cutaneous eruption known as herpes zoster, or "shingles." This expression of an intercostal neuritis was so marked some days ago that his parents brought him to the hospital. On admission he had been sick five days with a dull heavy pain over his lumbar region and with a slight rash on his hip. His temperature was ninety-nine, but otherwise his condition was about normal. This eruption consisted of raised vesicles which were elongated, so that the long diameter of the eruption corresponded to the course of the nerve trunks, which the eruption followed. These vesicles contained a certain amount of serum which escaped from time to time; but now they have disappeared and there only remains a red, irritative, sharply marked eruption.

This term herpes zoster, or zona, is not always applicable to the disease, for the trouble can follow the course of other nerve trunks besides the intercostal; for example, it can follow the course of the fifth facial nerve, involving even the safety of the eye upon that side of the head. The term "shingles," I might state, came to be applied to this disease from the Italian term for the disease, *shingula*, which means a zone or belt; hence the common English names for this trouble have all the same significance.

When it comes to the matter of treatment, in my experience the most satisfactory remedy is the tincture of the chloride of iron with the use, locally, of collodion containing morphia painted on the affected parts. In addition to this we must keep the bowels open, and if the pain grows too severe we must use anodynes. The diet should be nourishing and stimulating, including, if necessary, the use of alcohol in some form. It has been the habit here to use morphia in the collodion painted on locally, but on thinking the matter over, it seemed to me to be unnecessary to add the morphia, for, in the first place, it is not absorbed through the skin; it is frequently washed away by the breaking vesicles, and if held on the parts by the collodion, it is imbedded in the solid matter and can not be of service. I have found that the collodion alone has been as serviceable as the combination of collodion and morphine; its value is undoubtedly in the fact that it acts as a protective from the air and from the irritation which would come from friction of the affected parts

THE TREATMENT OF RHUS POISONING.

Our next patient is a man forty years of age who comes to us suffering that form of dermatitis known as rhus or "ivy poisoning." Four days prior to admission he was working in the country, where he was exposed to contact to poison ivy. On admission he had a temperature of ninety-nine degrees, but the rest of his physical examination was negative excepting for the severe dermatitis which he presented. It is a curious fact that this man, although exposed frequently before, has never had this disease until the present time. The man has had applications of "black wash," followed by the use of the ointment of the oxide of zinc, under which he has improved considerably.

The poison which causes this dermatitis is chemical in its nature, being an acid, having two sources, first, the ordinary three-leaved ivy and a small shrub, the rhus toxicondrium, which bears a resemblance among the ignorant to the sumac bush. This poison seems to vary in its power from time to time; it is probably given off more freely when the leaves are wet. Some persons are peculiarly susceptible to this poison; in their cases the mere walking in the vicinity of the ivy as it trails along some wall or low hedge is sufficient to produce the disease. Curiously enough, it is not readily communicated from person to person; we may find some one some time selfsacrificing enough to try inoculation, but it certainly must not be freely transmissible.

I do not have great faith in the value of drugs in these cases of rhus poisoning; like the case in erysipelas, the inflammation runs a certain course and seems to be unaffected by medication. Its course is limited; it never gets below a certain depth of the skin, and its duration is also definite. The value of drugs is to alleviate and soften the suffering of the patient; for this reason compresses of cold water or black wash containing a little carbolic acid or the bicarbonate of soda is indicated; practically I have found that the application of zinc ointment while the skin is still wet from the compress has an excellent effect. The fact that the disease is caused by an acid has led to the suggestion that this indicates the necessity of applying an alkaline wash; as far as I can determine this form of treatment has lead to no particularly brilliant results. It certainly has no specific action; in fact, the formation of blebs and their breaking, in itself, carries away a large part of any application. The treatment I have outlined gives in my hands the best results.

